# Mycosubtilin Produced by *Bacillus subtilis* ATCC6633 Inhibits Growth and Mycotoxin Biosynthesis of *Fusarium graminearum* and *Fusarium verticillioides*

**DOI:** 10.3390/toxins13110791

**Published:** 2021-11-09

**Authors:** Chenjie Yu, Xin Liu, Xinyue Zhang, Mengxuan Zhang, Yiying Gu, Qurban Ali, M. Sherif Ramzy Mohamed, Jianhong Xu, Jianrong Shi, Xuewen Gao, Huijun Wu, Qin Gu

**Affiliations:** 1 Key Laboratory of Integrated Management of Crop Diseases and Pests, Department of Plant Pathology, College of Plant Protection, Nanjing Agricultural University, Nanjing 210095, China; yuchenjie0501@163.com (C.Y.); xyzhang0661@163.com (X.Z.); zhangmengxuan0927@163.com (M.Z.); gyiying520@163.com (Y.G.); qurbanalirattar@webmail.hzau.edu.cn (Q.A.); gaoxw@njau.edu.cn (X.G.); hjwu@njau.edu.cn (H.W.); 2 Institute of Food Safety and Nutrition, Jiangsu Academy of Agricultural Science, Nanjing 210014, China; xinliu@jaas.ac.cn (X.L.); xujianhongnj@126.com (J.X.); jianrong63@126.com (J.S.); 3 School of Food and Biological Engineering, Jiangsu University, Zhenjiang 212013, China; 4 Department of Food Toxicology and Contaminant, National Research Centre of Egypt, Giza 12411, Egypt; sheriframzy4@gmail.com

**Keywords:** mycosubtilin, *Bacillus subtilis*, *Fusarium graminearum*, *Fusarium verticillioides*, biocontrol, mycotoxins

## Abstract

*Fusarium graminearum* and *Fusarium verticillioides* are fungal pathogens that cause diseases in cereal crops, such as Fusarium head blight (FHB), seedling blight, and stalk rot. They also produce a variety of mycotoxins that reduce crop yields and threaten human and animal health. Several strategies for controlling these diseases have been developed. However, due to a lack of resistant cultivars and the hazards of chemical fungicides, efforts are now focused on the biocontrol of plant diseases, which is a more sustainable and environmentally friendly approach. In the present study, the lipopeptide mycosubtilin purified from *Bacillus subtilis* ATCC6633 significantly suppressed the growth of *F. graminearum* PH-1 and *F. verticillioides* 7600 in vitro. Mycosubtilin caused the destruction and deformation of plasma membranes and cell walls in *F. graminearum* hyphae. Additionally, mycosubtilin inhibited conidial spore formation and germination of both fungi in a dose-dependent manner. *In planta* experiments demonstrated the ability of mycosubtilin to control the adverse effects caused by *F. graminearum* and *F. verticillioides* on wheat heads and maize kernels*,* respectively. Mycosubtilin significantly decreased the production of deoxynivalenol (DON) and B-series fumonisins (FB_1_, FB_2_ and FB_3_) in infected grains, with inhibition rates of 48.92, 48.48, 52.42, and 59.44%, respectively. The qRT-PCR analysis showed that mycosubtilin significantly downregulated genes involved in mycotoxin biosynthesis. In conclusion, mycosubtilin produced by *B. subtilis* ATCC6633 was shown to have potential as a biological agent to control plant diseases and *Fusarium* toxin contamination caused by *F. graminearum* and *F. verticillioides.*

## 1. Introduction

*Fusarium* is a cosmopolitan genus of filamentous ascomycete fungi. *Fusarium* species are toxin-producing plant pathogens that cause wilts, blights, and rots of various crops [1]. Fusarium head blight (FHB) disease caused by *Fusarium graminearum* affects many cereal crops and causes yield losses and reduction of grain quality [2,3]. *Fusarium verticillioides* causes seedling blight, stalk rot, and ear rot in maize [4,5]. *Fusarium* spp. produce a wide range of mycotoxins during disease development, and these are harmful to human and livestock health [6,7]. Trichothecenes and fumonisins are the significant and prominent mycotoxins produced by *Fusarium* species [8].

Deoxynivalenon (DON) and its acetylated derivatives (i.e., 3-DON, 15-DON) are common type B trichothecenes, mainly produced by *F. graminearum* [9,10]. DON is the most abundant mycotoxin detected in cereals, and high incidences and concentrations of DON have also been reported in feedstuffs [11]. DON is a potent inhibitor of protein synthesis, and it causes emesis, immune deficiency, and teratogenic effects in mammals [12]. Fumonisins, mainly synthesized by *F. verticillioides*, are the most common mycotoxins found in maize. Fumonisins are highly toxic, and their ingestion is associated with human toxicoses, including esophageal cancer, fetal neural tube defects, and hepatitis [13]. The control of *Fusarium* disease and mycotoxin contamination is challenging. The development of resistant varieties is time-consuming and labor-intensive. Chemical control is the main approach for preventing *Fusarium* disease, but the consequences of fungicide use include environmental pollution and the development of resistant fungal pathogens [14]. Control of *Fusarium* disease and mycotoxin accumulation requires alternative strategies and agents that are effective and environmentally friendly. The use of biological agents has attracted attention owing to their safety and efficacy [15].

Plant growth-promoting rhizobacteria (PGPR), which colonize the rhizosphere or plant root, can promote crop yield and suppress many plant pathogens [16]. Many PGPR-based commercial products are available, including Diegall, Galltrol-A, Zea-Nit, Quantum 4000, and Mycostop [17]. The genus *Bacillus* includes typical PGPR species and is the most promising genus for the development of biopesticides and biofertilizers. This is largely due to their ability to produce many antagonistic secondary metabolites with different structures. For example, *Bacillus subtilis* devotes approximately 4–5% of its genome for the synthesis of antibiotics. It can also produce abundant secondary metabolites with antimicrobial activity [18]. Among the active substances, lipopeptides (LPs), composed of a lipid tail linked to a linear or cyclic oligopeptide [19], were first reported to have a vital role in the biocontrol properties of *Bacillus*. Due to their structures, LPs from *Bacillus* have been divided into three families: iturin, fengycin (or pilpastatin), and surfactin [20,21]. Fengycins and iturins have strong in-vitro and in-vivo antagonistic activity toward fungal pathogens, including *F. graminearum* [14,20], *Botrytis cinerea* [22], *Sclerotinia sclerotiorum* [23], and *Candida albicans* [24,25]. The surfactin family contains powerful biosurfactants that kill bacteria by interfering with biological membrane integrity. Surfactins also have antiviral and anti-mycoplasma activities and contribute to biofilm formation and rhizosphere competition [26].

This study explored the mechanism underlying the antifungal activity of mycosubtilin and its effect on mycotoxin production. The results showed that the LP mycosubtilin from *B. subtilis* ATCC6633 can suppress the growth of *F. graminearum* and *F. verticillioides* as well as the biosynthesis of mycotoxins, including DON and fumonisins B1, B2, and B3. In addition, gene expression related to DON and B-series fumonisins (FBs) biosynthesis could be analyzed to show the negative influence of mycosubtilin on the DON and FB pathway. Therefore, we demonstrated that mycosubtilin has great potential as a biological agent, which can control plant diseases and mycotoxin contamination caused by *F. graminearum* and *F. verticillioides.*

## 2. Results

### 2.1. Mycosubtilin Produced by B. subtilis ATCC6633 Displayed In-Vitro Antagonistic Activity against F. graminearum and F. verticillioides

**Figure 1:**
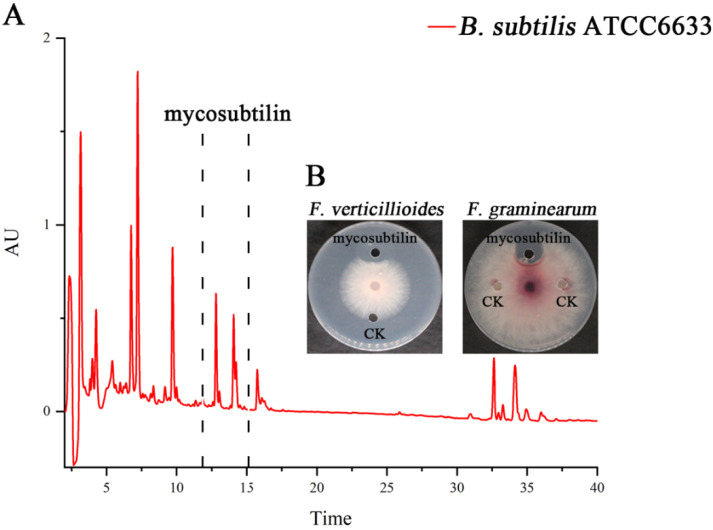
Purification and antifungal assay of mycosubtilin from *B. subtilis* ATCC6633. (**A**) Mycosubtilin was isolated from the crude extract of *B. subtilis* ATCC6633 using semipreparative reverse-phase HPLC. AU (absorbance unit). (**B**) Assay of antifungal activity using purified mycosubtilin against *F. graminearum* and *F. verticillioides*. CK (control), methanol.

**Figure 2:**
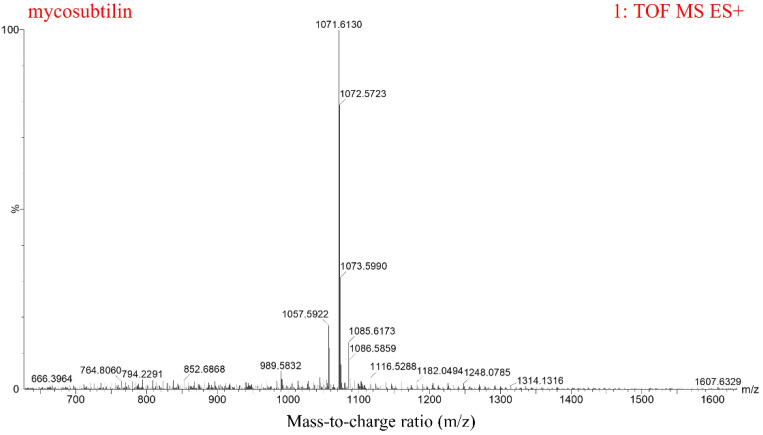
LC-MS analysis of purified mycosubtilin from *B. subtilis* ATCC6633. The ion peaks at 1057.6, 1071.6, and 1085.6 represent C_15_, C_16_, and C_17_ mycosubtilin homologues, respectively.

*B. subtilis* ATCC6633 has antifungal activity and produces two LPs, namely mycosubtilin and surfactin [27]. The crude antifungal extract was obtained by Amberlite Resin XAD16N extraction. Three peaks, from 11–15 min, for mycosubtilin were found, and it was purified by semipreparative HPLC (Figure 1A). The purified mycosubtilin was identified by LC-MS to confirm the existence of three peaks. There were molecular ion peaks ((M+H)^+^) for C_15_–C_17_ mycosubtilin at *m/z* 1057.6, 1071.6, and 1085.6, which was consistent with a previous report (Figure 2) [28]. The presence of fatty acid chains of varying lengths within mycosubtilin led to 14-Da differences in the molecular mass between several homologues of mycosubtilin [29]. The purity of mycosubtilin, which was used for further study, was found to be 94.3% via LC-MS analysis and normalization of peak areas. The antimicrobial assay showed that mycosubtilin had strong and long-lasting antagonistic activity against *F. graminearum* and *F. verticillioides*, and the inhibition zone had less than a 10% decrease after an additional 3 d of incubation (Figure 1B and Figure S1). The inhibitory effects of different concentrations of mycosubtilin against *F. graminearum* were determined. Mycosubtilin exerted antifungal activity in a dose-dependent manner at concentrations of 20–500 μg/mL (Figure S2). These results indicated that mycosubtilin inhibited the in-vitro growth of *F. graminearum* and *F. verticillioides*.

### 2.2. Ultrastructural Changes in F. graminearum Hyphae Caused by Mycosubtilin

**Figure 3:**
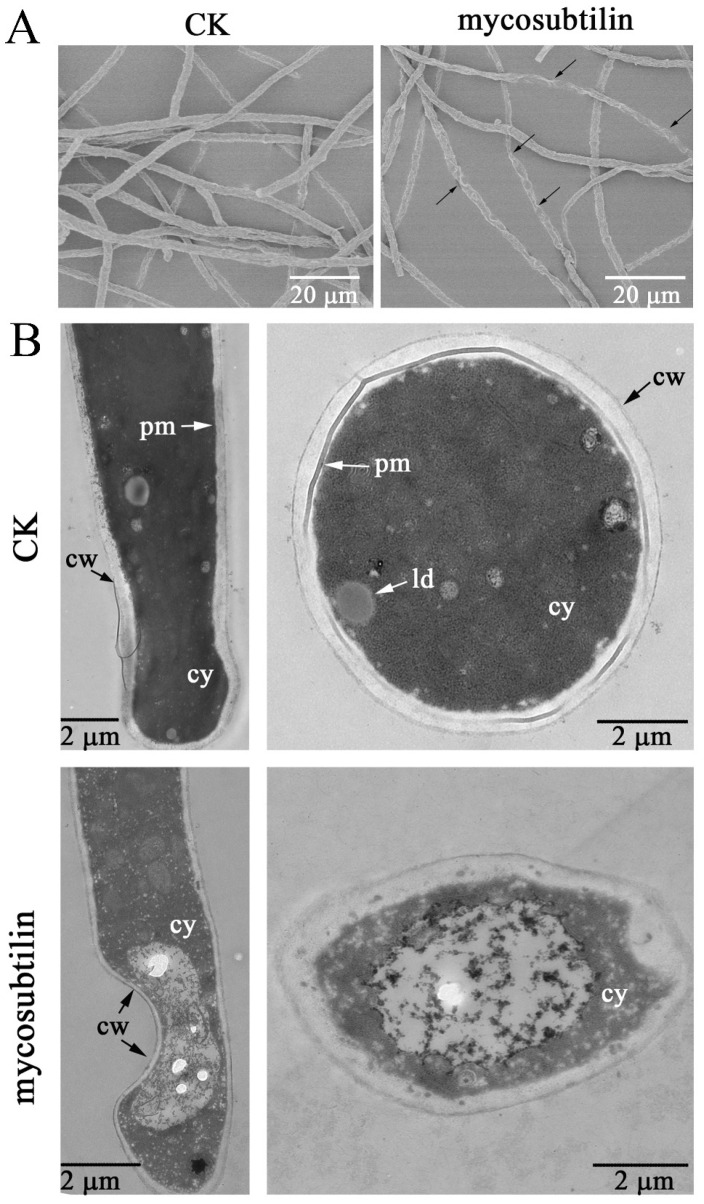
Morphology changes in *F. graminearum* hyphae induced by 20 μg/mL of mycosubtilin, detected via SEM (**A**) and TEM (**B**). CK, 25% (*vol*/*vol*) methanol. cw, cell wall; cy, cytoplasm; pm, plasma membrane; ld, lipid droplets.

Transmission electron microscopy (TEM) and scanning electron microscopy (SEM) observations were used to visualize the morphological changes in *F. graminearum* mycelia under mycosubtilin treatment. The SEM result showed that the untreated control hyphae of *F. graminearum* were dense, regular, plump, and cylindrical (Figure 3A). In contrast, after being treated with 20 μg/mL of mycosubtilin, destruction and deformation of fungal hyphae were observed, including curling, shrinkage, and breakdown (Figure 3A). Changes in the cellular morphology of *F. graminearum* were also observed using TEM. The untreated fungal hyphae had a well-defined cell wall, intact cell membrane, and uniformly distributed cytoplasm (Figure 3B). Compared with the control, fungal hyphae that had been treated with mycosubtilin showed impaired cellular integrity, irregular structure of the cell wall, and destruction of the cell membrane (Figure 3B).

### 2.3. Mycosubtilin Inhibited the Formation and Germination of Conidial Spores of F. graminearum and F. verticillioides

**Figure 4:**
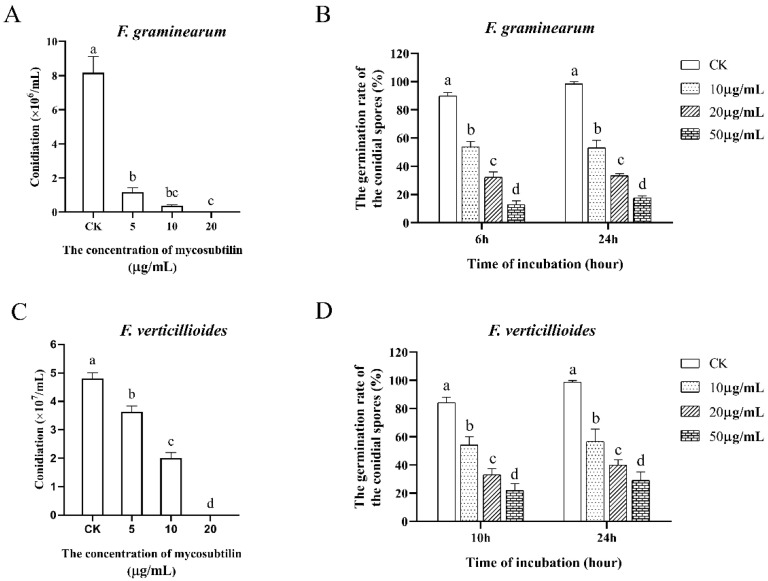
Effects of mycosubtilin on conidial formation and germination of *F. graminearum* (**A**,**B**) and *F. verticillioides* (**C**,**D**). CK, 25% (*vol*/*vol*) methanol. Data were analyzed by one-way ANOVA, followed by Duncan’s multiple range test. Line bars represent standard errors of three repeated experiments. Letters a, b, c, and d above the columns represent statistically significant differences (α = 0.05).

After investigating the hyphal changes induced by mycosubtilin, we studied its effect on fungal spore formation and germination, which are important for *F. graminearum* and *F. verticillioides* spread in plants. The formation of fungal spores was suppressed by mycosubtilin in a dose-dependent manner. When mycelia were treated with 20 μg/mL of mycosubtilin, few conidia were observed (Figure 4A,C). The expression analysis of conidiation-related genes (*fgsg_06774, fgsg_02471, fgsg_10166, fveg_05214, fveg_05267, fveg_05331*) was conducted to show that mycosubtilin could significantly affect conidiation at the transcriptional level (Figure S3). In the conidial germination assay, different concentrations of mycosubtilin decreased the germination rate of both fungi after treatment of 24 h. After treatment with 50 μg/mL of mycosubtilin for 24 h, the germination rate of *F. graminearum* and *F. verticillioides* was only 17.52 and 29.03%, respectively (Figure 4B,D). Additionally, mycosubtilin led to the conidia appearing swollen and dead and inhibited the elongation of germ tubes of both fungi (Figures S4–S6).

### 2.4. Mycosubtilin Reduced the Virulence of F. graminearum and F. verticillioides in Plants

**Figure 5:**
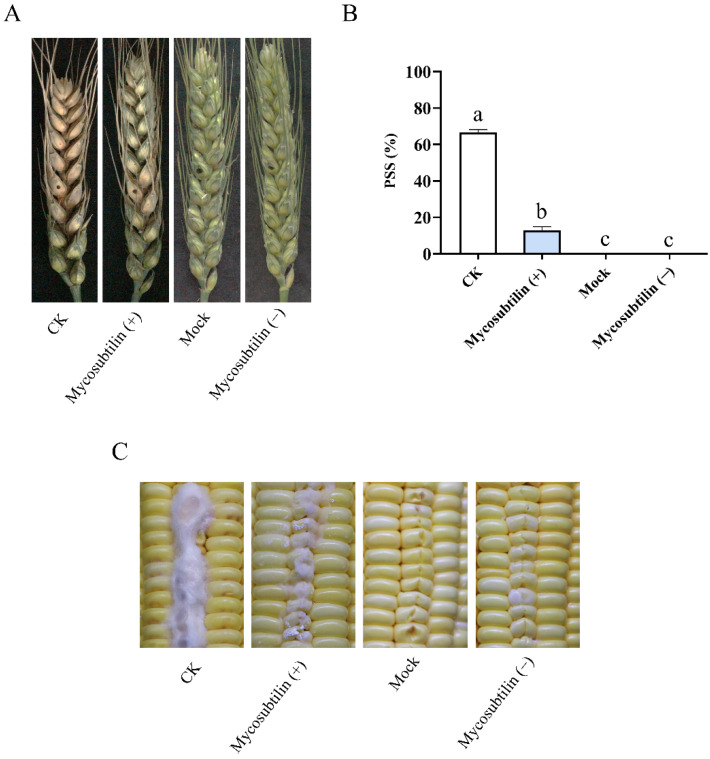
Effects of mycosubtilin on the pathogenicity of *F. graminearum* (**A**,**B**) and *F. verticillioides* (**C**) in plants. Mock, solution with 25% (*vol*/*vol*) methanol; CK, a conidial suspension with 25% methanol; Mycosubtilin (+), a conidial suspension with 100 μg/mL of mycosubtilin; Mycosubtilin (−), ddH_2_O containing 100 μg/mL of mycosubtilin. (**A**) *F. graminearum* PH-1 was used to infect wheat heads. Black dots on the wheat ears represent the inoculation site; (**B**) the pathogenicity of *F. graminearum* was measured by the average PSS. Data were analyzed by a one-way ANOVA, followed by Duncan’s multiple range test. Line bars represent standard errors of three repeated experiments. Letters a, b, and c above the columns represent statistically significant differences (α = 0.05); (**C**) *F. verticillioides* 7600 was used to infect maize kernels.

Mycosubtilin had strong in-vitro suppression activity against fungal pathogens. We therefore determined if it could reduce the pathogenicity of *F. graminearum* and *F. verticillioides* to plants. Wheat heads were inoculated with the conidia of *F. graminearum* containing 100 μg/mL of mycosubtilin. After 7 days, the diseased wheat kernels turned gray, while the healthy kernels remained green. The wheat heads treated with mycosubtilin remained healthy, and the average proportion of symptomatic spikelets (PSS) was reduced to 12.87% compared with the control. This indicated that mycosubtilin can reduce the pathogenicity of *F. graminearum* to wheat heads (Figure 5A,B). We also injected the conidia of *F. verticillioides* into wounded maize kernels and observed a reduction of aerial hyphae in the 100 μg/mL mycosubtilin treatment compared to the untreated control (Figure 5C). These results showed a 100 μg/mL mycosubtilin treatment could effectively inhibit the infection of *F. graminearum* and *F. verticillioides* on wheat and maize, respectively. Additionally, wheat heads and maize kernels treated with mycosubtilin alone remained healthy, suggesting that the use of mycosubtilin would not produce obvious detrimental side effects in plants.

### 2.5. Mycosubtilin Inhibited Mycotoxin Production of F. graminearum and F. verticillioides

DON produced by *F. graminearum* and the FBs produced by *F. verticillioides* are the most hazardous and the most studied mycotoxins. Consequently, a mycotoxin production assay was conducted to evaluate the effect of mycosubtilin on plural mycotoxins. Sterilized wheat/maize kernels were incubated with conidial suspensions of *F. graminearum*/*F. verticillioides* containing mycosubtilin. After 30 days of incubation, plural mycotoxins were extracted and quantified by LC-MS analysis. At a concentration of 50 μg/mL of mycosubtilin, the mycotoxin was detected by LC-MS, and after exposure to 100 μg/mL of mycosubtilin, fungal growth was completely inhibited, and the samples could not produce mycotoxin.

**Figure 6:**
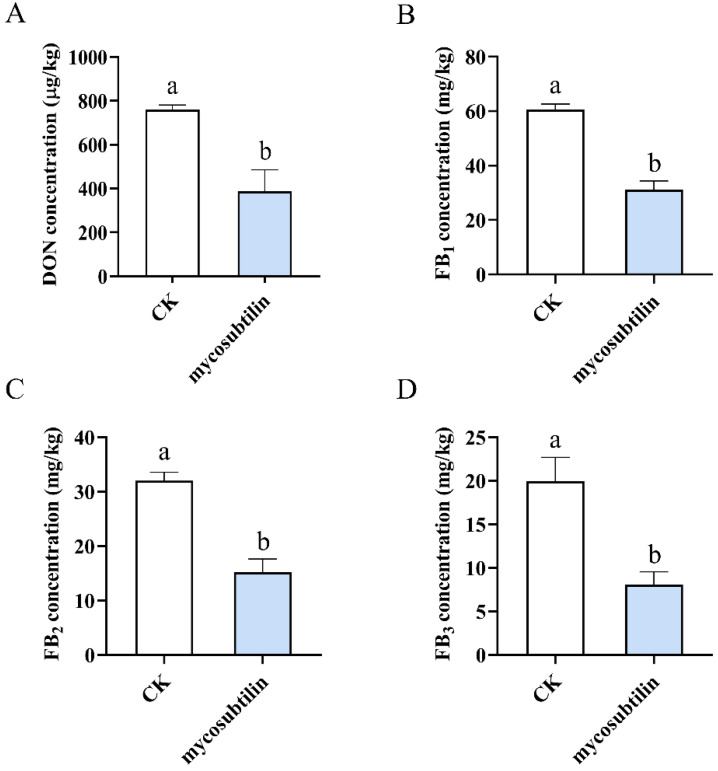
Effects of 50 μg/mL mycosubtilin treatment on mycotoxin biosynthesis. Content of DON (**A**) in wheat kernels and that of FB_1_ (**B**), FB_2_ (**C**), and FB_3_ (**D**) in maize kernels after 30 days of incubation, with or without mycosubtilin. CK, 25% (*vol*/*vol*) methanol. Data were analyzed by one-way ANOVA. Line bars represent standard errors of three repeated experiments. Letters a and b above the columns represent statistically significant differences (α = 0.05).

These results demonstrated that treatment with 50 μg/mL of mycosubtilin could reduce DON and FBs production by *F. graminearum* and *F. verticillioides,* respectively (Figure 6).

### 2.6. Mycosubtilin Downregulated the Expression Level of DON and FBs-Related Genes

**Figure 7:**
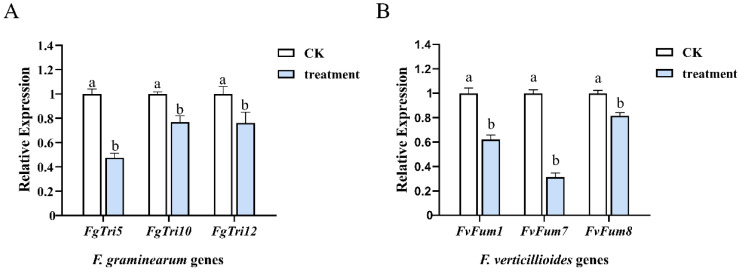
The expression level of DON-related genes of *F. graminearum* (**A**) and FBs-related genes of *F. verticillioides* (**B**). CK, 25% (*vol*/*vol*) methanol. Values were normalized to the levels of the actin gene as an internal reference. Data were analyzed by one-way ANOVA. Line bars represent standard errors of three repeated experiments. Letters a and b above the columns represent statistically significant differences (α = 0.05).

In *F. graminearum*, 15 *Tri* genes are responsible for the biosynthesis of trichothecene, while in *F. verticillioides*, the *Fum* gene cluster is involved in fumonisin production [11,30]. To study the mechanism underlying the reduction of DON and FBs production caused by mycosubtilin, the expression of *Tri* genes (*FgTri5*, *FgTri10,* and *FgTri12*) and *Fum* genes (*FvFum1*, *FvFum7,* and *FvFum8*) was analyzed. The expression level of six regulating genes was significantly downregulated by 50 μg/mL mycosubtilin treatment, suggesting that mycosubtilin can negatively influence the DON and FBs biosynthesis pathways (Figure 7).

## 3. Discussion

Lipopeptides (LPs) are important in *Bacillus* spp. biocontrol of phytopathogens [19]. They offer possible control of plant pathogen infections because they are selectively nontoxic, highly stable, and ecofriendly [31]. The fengycin and iturin families are strong inhibitors of fungal pathogens. Previous studies demonstrated that fengycin has antagonistic action against *F. graminearum* and *S. sclerotiorum* in vitro and in vivo [20,23]; bacillomycin D synthesized by *Bacillus velezensis* FZB42 had significant inhibitory activity against *F. graminearum* [29]. Mycosubtilin, a member of the iturin family, is characterized by a β-amino fatty acid linked to heptapeptide with a LDDLLDL chiral sequence in Asn-Tyr-Asn-Gln-Pro-Ser-Asn order [27]. Mycosubtilin exhibits antagonistic activities against *Bremia lactucae* [32], *Fusarium oxysporum* [33], and *S. cerevisiae* [34], suggesting its potential as an antifungal agent. However, the effects of mycosubtilin on *F. graminearum* or *F. verticillioides* have not yet been studied. In the present study, mycosubtilin was isolated from *B. subtilis* ATCC6633, and it showed a strong and durative inhibitory effect against *F. graminearum* and *F. verticillioides* in an antifungal assay (Figure 1 and Figure 2, Figures S1 and S2).

To explore the actions of mycosubtilin, SEM and TEM were used to observe the ultrastructural changes in the hyphae of *F. graminearum* PH-1. The SEM result indicated that 20 μg/mL of mycosubtilin had an adverse effect on the hyphal structure, while TEM analysis revealed that mycosubtilin can disrupt the cell wall and plasma membrane (Figure 3). These findings were consistent with previous research reporting that bacillomycin D, as another member of the iturin family, causes morphological changes in the hyphae and conidia of *F. graminearum* [29]. In other examples, LPs produced by *B. subtilis* B1 led to the plasmolysis and shrinkage of *Lasiodiplodia theobromae* [35]; iturins caused defects in the cell wall integrity of *Verticillium dahlia* [36], consistent with our results. The action of mycosubtilin damages hyphal cells by interacting with the ergosterol present in the membranes of fungi [37]. This suggests a possible reason for the morphological changes induced by mycosubtilin on the hyphal structure of *F. graminearum.*

*F. graminearum* and *F. verticillioides* infect crop plants by relying on sporulation and germination during their asexual life cycle. In the present study, fungal spore formation and germination were suppressed in vitro by mycosubtilin (Figure 4). To investigate the in-vivo inhibitory effect of mycosubtilin against these two pathogens, an *in planta* experiment was conducted, and it demonstrated that 100 μg/mL of mycosubtilin reduced the virulence of *F. graminearum* in wheat heads. Compared to the control, the average proportion of symptomatic spikelets (PSS) decreased sharply upon treatment with mycosubtilin (Figure 5A,B). These results were similar to the previous study wherein *F. graminearum*-infected wheat spikes were treated with 90 μg/mL of fengycin [20]. When the conidial spores of *F. verticillioides* were used to infect maize kernels, a distinct reduction of symptoms was observed relative to the control (Figure 5C), indicating the inhibitory effect of mycosubtilin on conidial spores. The adverse effects on hyphae caused by another iturin, bacillomycin D (90 μg/mL), reduced the infection of corn silks by *F. graminearum* hyphae, suggesting a potential function of mycosubtilin. Taken together, the pathogenicity tests of *F. graminearum* and *F. verticillioides* showed the substantial antagonistic activity of mycosubtilin against these fungi in vivo. The results suggest that mycosubtilin has potential for development as an antifungal agent. The dose of mycosubtilin used in the current study was similar to the concentrations of other LPs of *Bacillus* tested against *F. graminearum* [20,29].

However, the method of inoculating fungus together with mycosubtilin does not simulate a natural field situation. Additional experiments should be conducted to study the preventative effect of mycosubtilin against *F. graminearum* and *F. verticillioides.* Prior to field application, the protective effect of mycosubtilin on other wheat/maize cultivars against different strains of *F. graminearum*/*F. verticillioides* should also be studied.

DON is one of the most common mycotoxins associated with *F. graminearum*, and DON biosynthesis was inhibited by mycosubtilin (Figure 6A). Our results are supported by a previous study, which reported that iturin A, fengycin, and surfactin from *B. amyloliquefaciens* JCK-12 act together to decrease the yield of trichothecenes by *F. graminearum* [38]. However, Gu et al. reported that bacillomycin D can stimulate DON biosynthesis by activating the phosphorylation of *FgHOG1* and *FgMGV1*, which is in contrast to our findings [29]. We hypothesized that the distinct function of mycosubtilin and bacillomycin D in DON production is due to the different amino acid residues of heptapeptide. Fumonisins are the most abundant mycotoxins found in maize and are often associated with toxicoses in humans and livestock. FBs with a 20-carbon backbone have high toxicity and carcinogenicity. We also noted that mycosubtilin caused a large reduction in FBs production in maize kernels infected by *F. verticillioides* (Figure 6B–D)*,* which was similar to results showing that fengycin can downregulate the expression levels of *Fum1* and *Fum8* and inhibit the production of FB1 by *F. verticillioides* [39]. The qRT-PCR analysis showed that the expression level of *FgTri*/*FvFum* genes was downregulated after mycosubtilin treatment, demonstrating the inhibitory effect of mycosubtilin on DON and FBs biosynthesis at the transcriptional level (Figure 7). In summary, we report that mycosubtilin produced by *B. subtilis* ATCC6633 is a candidate compound for the control of the diseases and mycotoxin accumulation caused by *F. graminearum* and *F. verticillioides*.

## 4. Materials and Methods

### 4.1. Culture Conditions of Bacteria and Fungi

*B. subtilis* ATCC6633, *F. graminearum* PH-1, and *F. verticillioides* 7600 strains were stored in our laboratory. *B. subtilis* ATCC6633 strain was cultivated in Luria-Bertani (LB) medium at 37 °C, while the fungal species were cultivated in potato dextrose agar (PDA) at 25 °C. For the preparation of conidia suspension, fresh mycelia (50 mg) of *F. graminearum* PH-1 and *F. verticillioides* 7600 were inoculated in 20 mL liquid carboxymethyl cellulose (CMC) medium (1 g NH_4_NO_3_, 1 g KH_2_PO_3_, 0.5 g MgSO_4_·7H_2_O, 1 g yeast extract, 15 g carboxymethyl cellulose, and 1 L ddH_2_O) and cultured at 25 °C, 180 rpm, for 4 days (d).

### 4.2. Antifungal Activity Assay

The antagonistic activity of purified mycosubtilin was studied on a PDA plate. A 6-mm-diameter agar disk containing mycelia was placed at the center of a PDA plate. Subsequently, 7-mm-diameter wells were made with a sterilized bore at 3 cm from the center. Then, 10 μL of purified mycosubtilin was added to the wells and incubated at 25 °C for 6 d. The plates were then photographed, and the mycelial length of fungus was measured. The percentage of inhibition was calculated by using the following formula:

Inhibition rate (%) = [(C−T) × 100)/C]

where control (C) and treatment (T) are the mycelial length (cm) of the fungus from central mycelial plug to side edge of CK and mycosubtilin, respectively. The experiment was repeated three times, and three replicates were performed for each experiment.

### 4.3. Purification and Identification of Mycosubtilin from B. subtilis ATCC6633

A single colony of ATCC6633 was inoculated in 20 mL of LB medium and cultured at 37 °C for 24 h. Then, 2 mL of this culture was transferred to 200 mL of LB medium, followed by incubation for 48 h at 37 °C. After the first 24 h of incubation, 2 g of sterilized Amberlite Resin XAD16N (Lot BCCD6602, Sigma, St. Louis, MO, USA) was added to the culture to absorb compounds of molecular mass <3 kDa. The sample was then centrifuged at 10,000× rpm for 20 min, and the supernatant was discarded. The precipitates containing XAD16N were re-dissolved with 10 mL methanol and incubated at 37 °C for 4 h. Subsequently, the sample was filtered through double filter paper and concentrated using a rotary evaporator. The crude extract was obtained by suspending samples in methanol and filtration (0.22 μm, Nylon).

The crude extract was added to a semipreparative high-performance liquid chromatograph (HPLC, Waters, Milford, MA, USA) equipped with a reverse-phase column (ZORBAX SB-C18, 9.4 × 250 mm, 5 μm, Agilent, Palo Alto, CA, USA). The mobile phase comprised A (HPLC-grade acetonitrile with 0.1% trifluoroacetic acid) and B (Milli-Q water with 0.1% trifluoroacetic acid). The gradient elution procedure used 30–95% acetonitrile (ACN) for 30 min and then was held at 95% for 10 min. Mycosubtilin was eluted between 12–15 min. The detection was made under UV absorption at 214 nm, and the flow rate was 4 mL/min.

Purified mycosubtilin was verified via ultra-performance liquid chromatography-mass spectrometry (UPLC-MS, G2 QTof-XS, Waters, Milford, MA, USA) with a UPLC column (C_18_, 2.1 × 50 mm, 1.7 μm, Waters, Milford, MA, USA). Mobile phase A was ACN with 0.1% (*vol*/*vol*) formic acid, while mobile phase B was Milli-Q water (Millipore, Billerica, MA, USA). The samples were eluted from 2–98% ACN in 8 min and held at 98% for 2 min with the flow rate at 0.4 mL/min. The injection volume was 1 μL, and a photo-diode-array-detector was used to monitor the range of 200–400 nm. Mass spectrometry was carried out via an electrospray ionization (ESI) source operating in the positive ion mode. The MS parameters were as follows: source voltage 2.5 kV, collision energy 40 eV, and acquisition range 100–1500 *m/z.* The purified mycosubtilin was identified by comparing detected molecular ion peaks with those previously reported [28]. The purity of mycosubtilin was calculated, based on the peak area, as 94.3%.

### 4.4. Hyphal Morphological Observation via Scanning Electron Microscope (SEM) and Transmission Electron Microscope (TEM)

To observe the morphology changes of hyphae from *F. graminearum* PH-1 treated with mycosubtilin (20 μg/mL) at the ultrastructural level, TEM and SEM were used. Hyphae of *F. graminearum* were collected after 12 h treatment with mycosubtilin. For SEM, the samples were prefixed with 2.5% glutaraldehyde and followed by rinsing in triplicate using 100 mM phosphate buffer. Subsequently, the samples were postfixed for 3 h in 1% osmium tetroxide and dehydrated using an ethanol gradient. After that, the samples were coated with gold particles and observed via Hitachi S-3000N scanning electron microscope at voltage 5 kV (Hitachi, Tokyo, Japan). As for TEM analysis, the prefixed cells were embedded in Epon 812, sectioned using an ultra-microtome, and examined with a Hitachi H-600 transmission electron microscope.

### 4.5. The Assay of Conidia Formation and Germination

For the spore formation assay, the mycelia of *F. graminearum* (or *F. verticillioides*) was inoculated in CMC medium containing different concentrations of mycosubtilin (5, 10, and 20 μg/mL), and 25% (*vol*/*vol*) methanol was used as the control. After 3 d of incubation at 25 °C and 180 rpm, the number of conidia was measured using a hemacytometer. The experiment was repeated three times with three replicates.

To determine the germination rate of fungal spores, 1 mL of conidial suspension (10^6^ conidia/mL) with different concentrations of mycosubtilin (10, 20, and 50 μg/mL) were cultured at 25 °C and 180 rpm. The incubation time for *F. graminearum* is 6 and 24 h, while that for *F. verticillioides* is 10 and 24 h. A spore was considered germinated if the germ tube reached at least half the length of the spore. The germination rate was quantified by counting at least 200 conidia for each replication by microscopy. As the control, 25% (*vol*/*vol*) methanol was used. The experiment was repeated three times with three replicates.

Germination and conidial morphology of each sample were examined using the DIC mode of an OLYMPUS-IX71 inverted microscope (Tokyo, Japan). The conidia that had been treated with 10 μg/mL mycosubtilin for 3 d were assayed using propidium iodide (Solarbio, Beijing, China). Fungal cells were imaged using an OLYMPUS-IX71 inverted microscope, and those with damaged membranes showed red fluorescence.

### 4.6. Pathogenicity Assay

To detect the effect of mycosubtilin on the pathogenicity of *F. graminearum* and *F. verticillioides* in wheat heads and maize kernels, corresponding greenhouse experiments were conducted. The experiment consisted of four treatments: (1) Mock, inoculated with 10 μL of 25% (*vol*/*vol*) methanol only; (2) CK, inoculated with 10 µL conidial suspension with 25% methanol; (3) Mycosubtilin (+)*,* treated with 10 µL conidial suspension containing 100 μg/mL mycosubtilin; and (4) Mycosubtilin (−), treated with 10 µL ddH_2_O containing 100 μg/mL mycosubtilin. The concentration of conidial suspension used in greenhouse experiments was 10^6^ conidia/mL.

For wheat heads, wheat that reached the anthesis stage was used. The fifth spikelet from the base of the spike was drop inoculated with different solutions of treatment. The plants were kept in the greenhouse with condition as follows: 25 °C, 100% humidity, and 12 h of the daylight period. After 7 d, different samples were photographed, and the average proportion of symptomatic spikelets (PSS) was measured [40]. The experiment was repeated three times, and fourteen replicates were performed for each treatment.

For maize kernels, their surface was first sterilized by a spray of 75% ethanol and ddH_2_O. After that, the maize kernels were scratched using sterilized toothpicks and inoculated under different treatments. Three replicates were used for each treatment. Maize kernels were incubated under 25 °C, 100% humidity, for 7 d, in a 12-h daylight period [41]. The experiment was repeated three times.

### 4.7. Determination of Mycotoxin Production

A total of 50 g of detached wheat (or maize) kernels were sterilized and inoculated with 1 mL conidial suspension (10^6^ conidial/mL) of *F. graminearum* (or *F. verticillioides*) containing 50 μg/mL purified mycosubtilin. As the control, 1 mL conidial suspension with 25% (*vol*/*vol*) methanol was used. After incubation at 25 °C for 30 d, mycotoxin was extracted as described previously [42,43,44] and analyzed using a HPLC-mass spectrometer/mass spectrometer (HPLC-MS/MS) system (Shimadzu 30A LC system coupled to a Triple Quad 6500 plus, Sciex, Framingham, MA, USA) to determine the amount of mycotoxin in the corresponding sample. The experiment was repeated thrice, and three replicates were performed for each treatment.

### 4.8. RNA Extraction and Expression Analysis by qRT-PCR

For total RNA extraction, mycelia of *F. graminearum* PH-1 (or *F. verticillioides* 7600) were inoculated in a 50-mL flask containing 20 mL of potato dextrose broth (PDB) at 25 °C for 36 h. Either 50 μg/mL mycosubtilin or 25% methanol (set as a control treatment) was added into the flask for another 2 h. Followed by the manufacturer’s guidelines, the total RNA of each sample was extracted from the harvested mycelia using the TaKaRa RNAiso reagent (TaKaRa Biotechnology Co., Ltd., Dalian, China). Reverse transcription was conducted by using HiScript III RT SuperMix (Vazyme Biotech Co., Ltd., Nanjing, China). The expression level of *Tri* and *Fum* genes was investigated in a 7500 fast real-time PCR detector by using HiScript II One Step qRT-PCR SYBR Green Kit (Vazyme Biotech Co., Ltd. Nanjing, China). The conditions of qRT-PCR consisted of hold at 95 °C for 30 s, followed by 40 cycles of 5 s at 95 °C and 30 s at 60 °C. The *FgActin* and *FvActin* gene were amplified as housekeeping genes for normalization. The expression analysis was carried out with the ΔΔCT method using the 7500 system SDS software. Primers used for qRT-PCR are listed in Table S1. The experiment was repeated thrice independently, with three replicates for each treatment.

## Data Availability

Data are available upon request; please contact the contributing authors.

## Supplementary Materials

The following are available online at https://www.mdpi.com/article/10.3390/toxins13110791/s1, Table S1: Primers used in this study; Figure S1: In the in vitro antifungal assay, percentage inhibition of mycosubtilin against *F. graminearum* and *F. verticillioides* after incubation of 3 days and 6 days was calculated. Data were analyzed by one-way ANOVA. Line bars represent standard errors of three repeated experiments. Same letter of a above the columns represents no statistically significant differences (α = 0.05); Figure S2: In the in vitro antifungal assay, percentage inhibition of mycosubtilin (20, 50, 100, 250, and 500 μg/mL) against *F. graminearum* after incubation of 3 days and 6 days was calculated. Data were analyzed by one-way ANOVA, followed by Duncan’s multiple range test. Line bars represent standard errors of three repeated experiments. Letters a, b and c above the columns represent statistically significant differences (α = 0.05), while similar letters ab and bc represent no statistically significant differences; Figure S3: The expression level of conidiation-related genes *(fgsg_06774*, *fgsg_02471*, *fgsg_10166*, *fveg_05214*, *fveg_05267*, *fveg_05331*) of *F. graminearum* (A) and *F. verticillioides* (B). CK (control), 25% (*vol/vol*) methanol. Treatment, 50 μg/mL mycosubtilin. Values were normalized to the levels of the actin gene as an internal reference. Data were analyzed by one-way ANOVA. Line bars represent standard errors of three repeated experiments. Letters a and b above the columns represent statistically significant differences (α = 0.05); Figure S4: Morphology changes in conidia of *F. graminearum* and *F. verticillioides* induced by 10 μg/mL of mycosubtilin, detected by inverted microscope. CK, 25% (*vol/vol*) methanol; Figure S5: Inhibitory activity of different concentrations of mycosubtilin (10, 20 and 50 μg/mL) on conidial germination of *F. graminearum* and *F. verticillioides*, detected by inverted microscope. CK, 25% (*vol/vol*) methanol; Figure S6: Viablity of *F. graminearum* (A) and *F. verticillioides* (B) conidia after treatment with 10 μg/mL of mycosubtilin for 3 days. Fungal cells with damaged membranes showed red fluorescence. CK, 25% (*vol/vol*) methanol.
