# Supplementary material for: Mycosubtilin Produced by Bacillus subtilis ATCC6633 Inhibits Growth and Mycotoxin Biosynthesis of Fusarium graminearum and Fusarium verticillioides
Source: Toxins (Basel). 2021 Nov 9;13(11):791. doi: 10.3390/toxins13110791 (PMC8620035; doi:10.3390/toxins13110791)
Supplement: Supplementary file 1 [file toxins-13-00791-s001.zip › toxins-1368530 - Supplementary -Final.pdf]

toxins

Supplementary Materials

Mycosubtilin Produced by *Bacillus Subtilis* ATCC6633 Inhibits Growth and Mycotoxin Biosynthesis of *Fusarium graminearum* and *Fusarium verticillioides*

Chenjie Yu [1,†], Xin Liu [2,3,†], Xinyue Zhang [1], Mengxuan Zhang [1], Yiying Gu [1], Qurban Ali [1], M Sherif Ramzy Mohamed [4], Jianhong Xu [2,3], Jianrong Shi [2,3], Xuewen Gao [1], Huijun Wu [1], and Qin Gu [1,*]

[1] Key Laboratory of Integrated Management of Crop Diseases and Pests, Department of Plant Pathology, College of Plant Protection, Nanjing Agricultural University, Nanjing 210095, China; yuchenjie0501@163.com (C.Y.); xyzhang0661@163.com (X.Z.); zhangmengxuan0927@163.com (M.Z.); gyiying520@163.com (Y.G.); qurbanalirattar@webmail.hzau.edu.cn (Q.A.); gaoxw@njau.edu.cn (X.G.); hjwu@njau.edu.cn (H.W.)
[2] Institute of Food Safety and Nutrition, Jiangsu Academy of Agricultural Science, Nanjing 210014, China; xinliu@jaas.ac.cn (X.L.); xujianhongnj@126.com (J.X.); jianrong63@126.com (J.S.)
[3] School of Food and Biological Engineering, Jiangsu University, Zhenjiang 212013, China
[4] Department of Food Toxicology and Contaminant, National Research Centre of Egypt, Giza 12411, Egypt; sheriframzy4@gmail.com (M.M.)
* Correspondence: guqin@njau.edu.cn
† These authors contributed equally to this work.

Table S1. Primers used in this study.

Primer name	Sequence (5′ to 3′)
Tri5-F	AGTGCTACGGATAAGGTTCA
Tri5-R	GGCAAAGGTGTCCAAAGAG
Tri10-F	CCTTGTGATGCCAAATGTC
Tri10-R	GAGACGAGCCTGTTGATGT
Tri12-F	ATCCCTCTACAGCAACCAG
Tri12-R	ATCGGAATCTCCCTCAAGT
FUM1-F	TGCTGCCCTGTATCACAACCA
FUM1-R	AATGTGCGCTTGATCCAGTT
FUM7-F	GCATGGAGAGACAAGTTGCA
FUM7-R	TCTGATGAAACTGGGCTTCGT
FUM8-F	GCGACGGTCCAAATGATTAA
FUM8-R	TCAATGAATGAGGCACTGCT
Fgsg_06774-F	TCCAGCAAGCTATAACGG
Fgsg_06774-R	GTTTTGCGGAATAGAGCT
Fgsg_02471-F	ACCTCAACTATCAGCAGAGT
Fgsg_02471-R	GTACTCGGATAAGGGCTCCA

Fgsg_10166-F	AACGCCTACCACGGTTCACT
Fgsg_10166-R	TTGACGAACCCTATATCTCC
Fveg_05214-F	TGCCATTCTTCAACGGAATA
Fveg_05214-R	GTGATACTCTGTGAAGGGCG
Fveg_05267-F	GAAGATGAGGATTTCTCTGG
Fveg_05267-R	GCTGAGAGTCTCGAGACTAT
Fveg_05331-F	TAGTCCAATCGAAGCACAAC
Fveg_05331-R	AGGTTCTTGGGGTCCTGGAA
FgActin-F	ATCCACGTCACCACTTTCAA
FgActin-R	TGCTTGGAGATCCACATTTG
FvActin-F	TGCTCCTGAGGCTCTCTTCCA
FvActin-R	AAGCAAGAATAGAACCACCGA

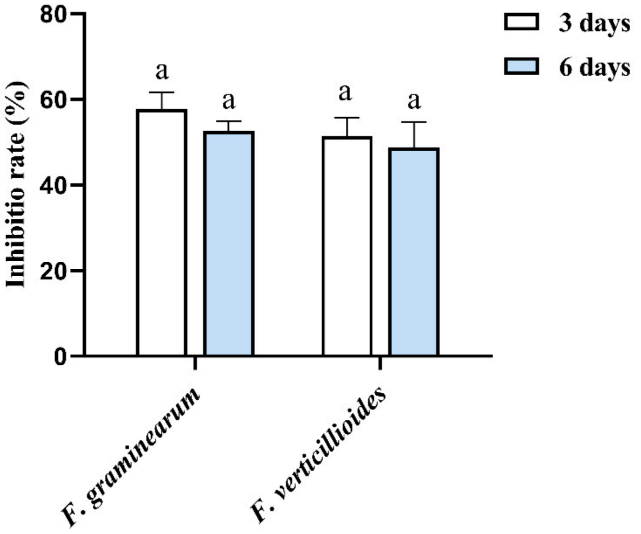

Figure S1. In the *in vitro* antifungal assay, percentage inhibition of mycosubtilin against *F. graminearum* and *F. verticillioides* after incubation of 3 days and 6 days was calculated. Data were analyzed by one-way ANOVA. Line bars represent standard errors of three repeated experiments. Same letter of a above the columns represents no statistically significant differences (α= 0.05).

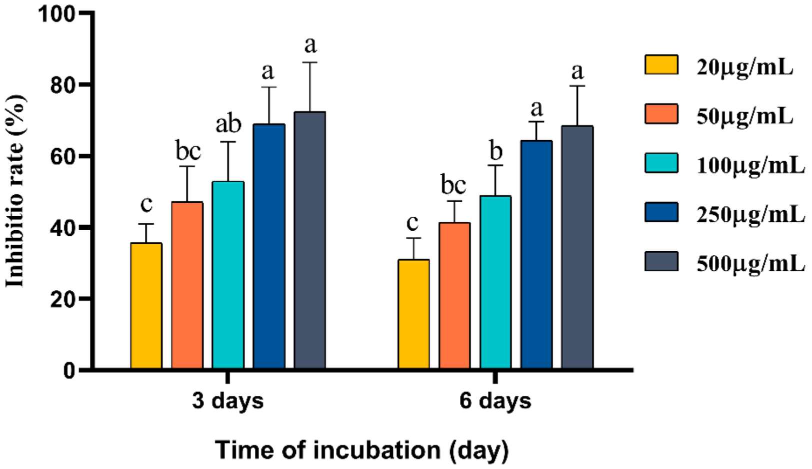

Figure S2. In the *in vitro* antifungal assay, percentage inhibition of mycosubtilin (20, 50, 100, 250, and 500 µg/mL) against *F. graminearum* after incubation of 3 days and 6 days was calculated. Data were analyzed by one-way ANOVA, followed by Duncan's multiple range test. Line bars represent standard errors of three repeated experiments. Letters a, b and c above the columns represent statistically significant differences (α= 0.05), while similar letters ab and bc represent no statistically significant differences.

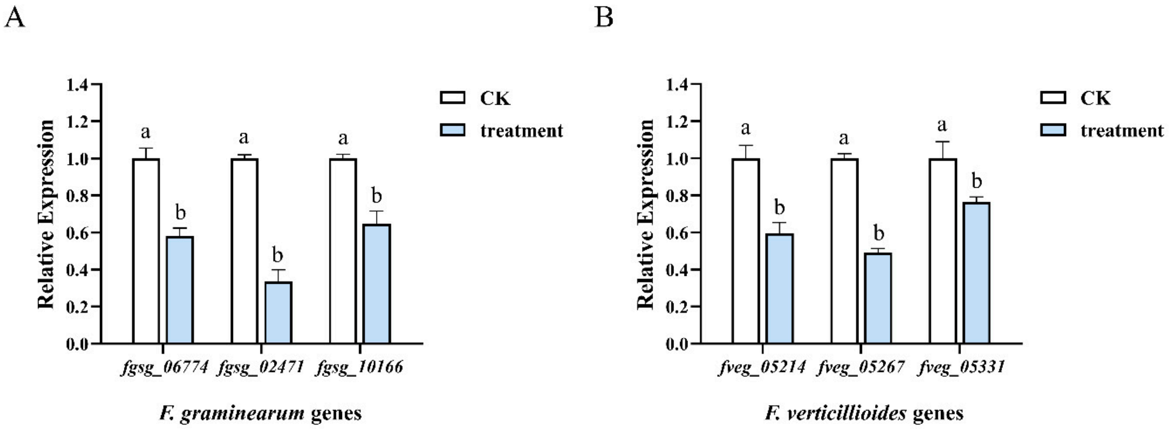

Figure S3. The expression level of conidiation-related genes (*fgsg_06774*, *fgsg_02471*, *fgsg_10166*, *fveg_05214*, *fveg_05267*, *fveg_05331*) of *F. graminearum* (A) and *F. verticillioides* (B). CK (control), 25% (vol/vol) methanol. Treatment, 50 µg/mL mycosubtilin. Values were normalized to the levels of the actin gene as an internal reference. Data were analyzed by one-way ANOVA. Line bars represent standard errors of three repeated experiments. Letters a and b above the columns represent statistically significant differences (α= 0.05).

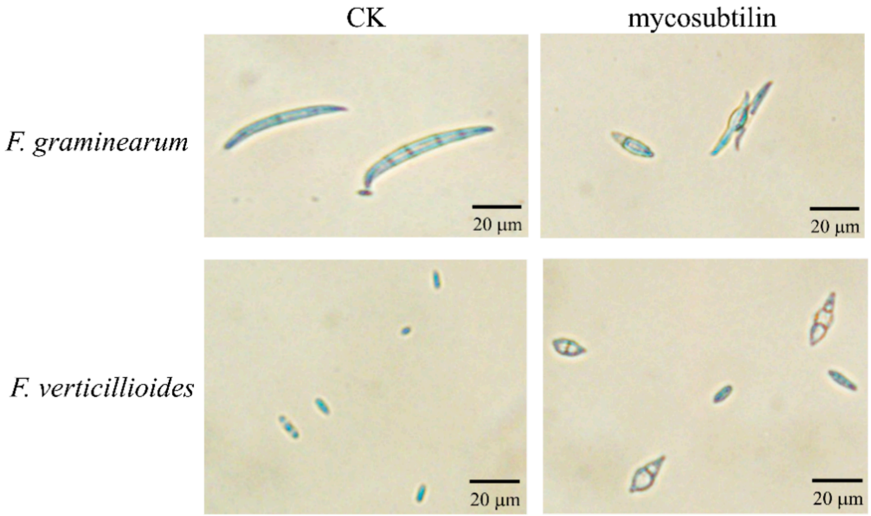

Figure S4. Morphology changes in conidia of *F. graminearum* and *F. verticillioides* induced by 10 μg/mL of mycosubtilin, detected by inverted microscope. CK, 25% (vol/vol) methanol.

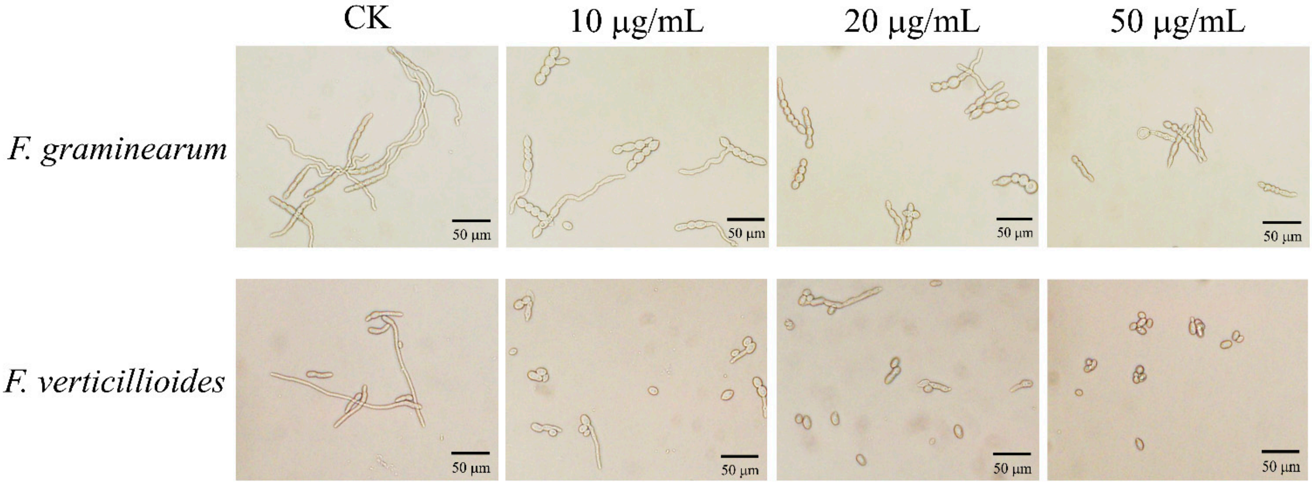

Figure S5. Inhibitory activity of different concentrations of mycosubtilin (10, 20 and 50 μg/mL) on conidial germination of *F. graminearum* and *F. verticillioides*, detected by inverted microscope. CK, 25% (vol/vol) methanol.

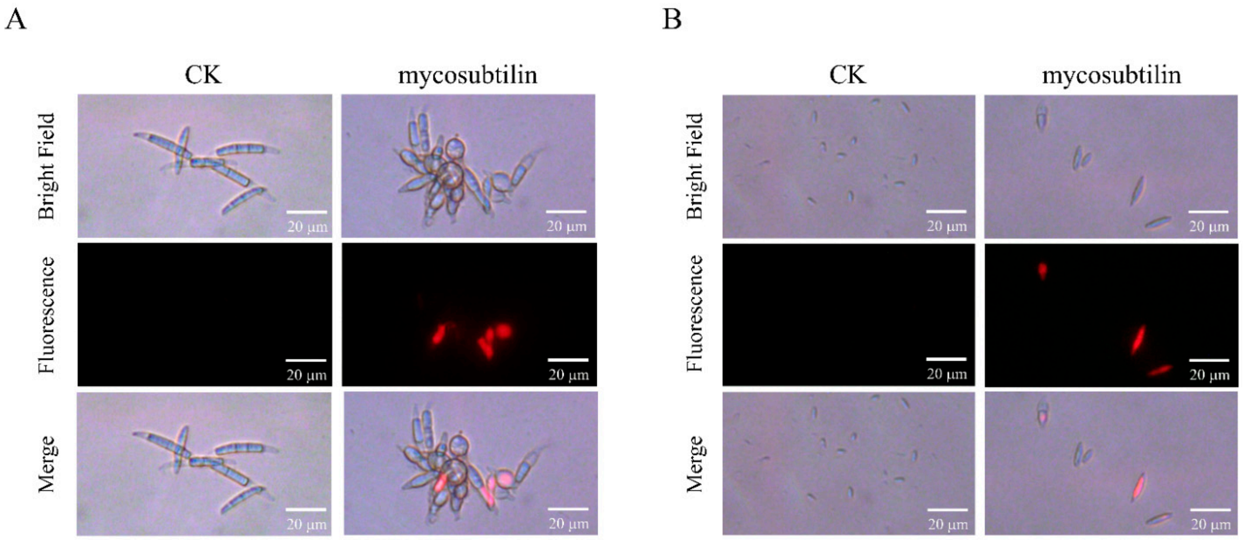

Figure S6. Viablity of *F. graminearum* (A) and *F. verticillioides* (B) conidia after treatment with 10 μg/mL of mycosubtilin for 3 days. Fungal cells with damaged membranes showed red fluorescence. CK, 25% (vol/vol) methanol.
